# Small Intestinal Absorption of Methylsulfonylmethane (MSM) and Accumulation of the Sulfur Moiety in Selected Tissues of Mice

**DOI:** 10.3390/nu10010019

**Published:** 2017-12-25

**Authors:** Thomas Wong, Richard J. Bloomer, Rodney L. Benjamin, Randal K. Buddington

**Affiliations:** 1 Center for Nutraceutical and Dietary Supplement Research, School of Health Studies, The University of Memphis, Memphis, TN 38152, USA; thomaslwong@gmail.com (T.W.); rbloomer@memphis.edu (R.J.B.); 2 Bergstrom Nutrition, 1000 W. 8th Street, Vancouver, WA 98660, USA; RBenjamin@bergstromnutrition.com

**Keywords:** methylsulfonylmethane, DMSO2, methyl sulfone, dimethyl sulfone, supplement, posttranslational

## Abstract

The principal dietary sources of sulfur, the amino acids methionine and cysteine, may not always be consumed in adequate amounts to meet sulfur requirements. The naturally occurring organosulfur compound, methylsulfonylmethane (MSM), is available as a dietary supplement and has been associated with multiple health benefits. Absorption of MSM by the small intestine and accumulation of the associated sulfur moiety in selected tissues with chronic (8 days) administration were evaluated using juvenile male mice. Intestinal absorption was not saturated at 50 mmol, appeared passive and carrier-independent, with a high capacity (at least 2 g/d-mouse). The ^35^S associated with MSM did not increase in serum or tissue homogenates between days 2 and 8, indicating a stable equilibrium between intake and elimination was established. In contrast, proteins isolated from the preparations using gel electrophoresis revealed increasing incorporation of ^35^S in the protein fraction of serum, cellular elements of blood, liver, and small intestine but not skeletal muscle. The potential contributions of protein synthesis using labeled sulfur amino acids synthesized by the gut bacteria and posttranslational sulfation of proteins by incorporation of the labeled sulfate of MSM in 3′-phosphoadenosine 5′-phosphosulfate (PAPS) and subsequent transfer by sulfotransferases are discussed.

## 1. Introduction

Sulfur represents ~0.3% of total body mass and is the 7th most abundant element in the body. The majority of dietary sulfur is provided by the sulfur amino acids methionine and cysteine, with an estimated requirement for young men of ~13 mg/day per kg body weight [1]. Although there are numerous other natural sources of dietary sulfur, the lower proportions of natural foods in the Western diet has led to concerns of inadequate intake and possible need for supplemental sources [2]. Sulfur is involved in a number of key metabolic pathways such as carbohydrate metabolism, protein synthesis, redox balance and detoxification. Insufficient or marginal intake can result in the initiation and progression of several inflammatory and degenerative changes associated with a large number of pathologies. Evidence continues to grow supporting the vital role dietary sulfur plays in fundamental mechanisms of metabolism and physiology [2]. Methylsulfonylmethane (MSM), also known by several other names (DMSO2, methyl sulfone and dimethyl sulfone), is a naturally occurring source of sulfate available in different fruits, vegetables, grains and animal tissues and considered to provide health benefits when used to supplement the diet [3]. MSM obtained Generally Recognized as Safe (GRAS) status in 2007 and has been used as a human dietary supplement for animals and humans since the early 1980’s [4,5]. The global market is expected to reach over US$10 billion by 2023 (https://www.credenceresearch.com/press/global-methylsulfonylmethane-market) [6] because of the increasing body of evidence supporting health benefits.

Supplemental sulfur may be necessary when the diet does not provide adequate amounts of sulfur amino acids, particularly for strict vegetarians [7] and when the sulfur requirement is higher, such as during disease states [8] and pregnancy and fetal development [9]. Although mammals are unable to use the supplemental sources of sulfur to synthesize methionine and other sulfur amino acids, inorganic sulfur as sulfate has vital roles [9]. Numerous proteins and other non-protein molecules require sulfation. This process involves the transfer of sulfate from 3′-phosphoadenosine 5′-phosphosulfate (PAPS) to various substrates by sulfotransferases [10]. The primary source of the sulfate used in this pathway is cysteine. Alternative sources of the sulfate can spare cysteine for other purposes, such as synthesis of glutathione.

Despite the presence of MSM in numerous natural foods and growing use as a dietary supplement, definitive data are lacking for intestinal absorption and whether MSM serves as a sulfur donor. Based on in vivo studies with human subjects and animal models, oral MSM is rapidly distributed throughout the body, including passing through the blood brain barrier [11] but is rapidly cleared and mostly via the urine, with some evidence of retention with chronic administration [12]. However, the potential of MSM to serve as sulfur source for protein synthesis and thereby be incorporated into tissues remains uncertain. This project had two objectives that address deficits in understanding the utilization of dietary MSM: (1) directly measure intestinal absorption of MSM and (2) assess if there is progressive accumulation of the sulfur moiety in blood and selected tissues and the associated protein fractions when administered to mice over 8 days with samples collected after 2, 5 and 8 days of administration.

## 2. Materials and Methods

### 2.1. Animals and Their Care

Juvenile (4–6 weeks) male C57B6 mice were group housed in standard cages (3–5 mice per cage) with constant access to a standard rodent chow and water. All aspects of the project involving care and use of the mice had been approved the University of Memphis Institutional Animal Care and Use Committee (protocol #0780). 

### 2.2. Small Intestinal Absorption of MSM

Following an established method [13], rates of MSM absorption (nmol/mg-min) were measured at 50 mM and tracer concentration (0.03 mM) using intact sleeves of everted proximal and distal small intestine prepared from two mice. From each region four tissue sleeves were prepared alternating between 50 mM and tracer. The tissues were incubated for 2 min in aerated and stirred mammalian Ringers with or without 50 mM unlabeled MSM (OptiMSM^®^; Bergstrom Nutrition Vancouver, WA, USA). Tracer concentration of ^35^S MSM (Moravek Inc., Brea, CA, USA) was added (0.03 mM) to each tube with tracer ^3^H polyethylene glycol (4000 MW) also added to correct for MSM associated with the adherent fluid and not accumulated within the tissue. After the incubation, the tissues were gently blotted, removed and wet-weight recorded, solubilized (Soluene^®^-350; Perkin-Elmer, Waltham, MA, USA), scintillant was added (UltimaGold; Perkin-Elmer, Waltham, MA, USA ) and the accumulated ^35^S labeled MSM and ^3^H polyethylene glycol were measured by liquid scintillation counting (LSC). The associated radioactivity was used to calculate MSM accumulation per min per mg wet tissue mass.

### 2.3. Tissue Accumulation of MSM

To determine if there was progressive accumulation of the sulfur moiety of MSM, ^35^S labeled MSM was provided to mice for 2 (*n* = 4), 5 (*n* = 5) and 8 days (*n* = 5) using pureed sweet potato, which is rapidly accepted and completely consumed by mice, was used as the vehicle. Once the mice in each cage learned to rapidly consume the vehicle from a metal pan (equivalent to 1 gm per mouse in 5 min), the ^35^S labeled MSM was added to the sweet potato to provide a dose of 10 μCi (0.6 μM; 5.5 μg) per day for each of the mice in each cage. Addition of the labeled MSM did not affect consumption of the vehicle with the full dose consumed within 3–5 min after presentation.

The mice were euthanized (CO_2_ asphyxiation) 18–24 h following the last dose, blood was collected from the inferior vena cava and serum was separated by centrifugation. The liver, small intestine and skeletal muscle from both hind limbs were collected, weighed and homogenized in 4 mL of ice cold Ringers after which 1 mL of 5X RIPA buffer (50 mM TrisHCl pH7.4, with 150 mM NaCl, 2 mM EDTA, 1% NP-40, 0.1% SDS) was added. Samples of serum (8 μL) and aliquots of the homogenized tissues (100 μL) were added to 0.5 mL of tissue solubilizer, incubated overnight at 60 °C, scintillant was added and radioactivity was measured by LSC. Protein content of the serum and tissue homogenates were measured using the Bio-Rad protein assay dye reagent (Bio-Rad, Hercules, CA, USA) and compared with a bovine serum albumin standard. Gel electrophoresis was used to separate the proteins in the serum and homogenates. 8 μL of serum were added to 2 μL of 5X loading buffer (250 mM TrisHCl pH6.8, 10% SDS, 30% glycerol, 5% 2-mercaptoethanol, 0.02% bromophenol blue) and 80 μL aliquots of each homogenate were added to 20 μL of 5X loading buffer, before heating for 10 min in a boiling water bath for 10 min. The entire serum preparation was loaded into a single lane whereas two 50 μL portion of the prepared homogenate were loaded into each of two lanes of 4–20% Mini-PROTEAN^®^ TGX™ Precast Protein Gels with 50 µL wells (Bio-Rad, Hercules, CA, USA). A protein molecular weight marker was added to one lane. Electrophoresis with a running buffer of Tris-Glycine-SDS (25 mM Tris, 192 mM glycine and 0.1% SDS, pH approx. 8.6) continued until the 10 kD marker was ~1 cm above the end of the gel. The lanes were cut out ~0.5 cm below the well so that only protein that had migrated into the gel was recovered. The gel segments with the lanes were homogenized in the solubilizer, left overnight at 60 °C, the scintillant was added and ^35^S was measured by LSC.

### 2.4. Statistics

ANOVA was used to detect temporal effects on accumulation of ^35^S in homogenates and gels. *t*-tests were used to detect differences between specific days for the accumulation of ^35^S in homogenates and gels for each tissue, using a critical value of *p* < 0.05. 

## 3. Results

### 3.1. Small Intestine MSM Absorption

Rates of absorption (nmol/mg-min) did not differ between the proximal and distal regions of the small intestine, whether measured at 50 mM or at tracer concentration (Table 1). Accumulation of tracer MSM by the tissues did not differ in the presence or absence of 50 mmol unlabeled MSM (0.0073 ± 0.0003 vs. 0.0068 ± 0.0003; *p* = 0.13; pooled data for proximal and distal regions). These findings indicate absorption of MSM does not compete for transporters and is driven by an inwardly driven concentration gradient. As an additional indicator, ratios were calculated using adjacent tissues for amount of MSM absorbed at 50 mmol relative to tracer only. The value of 1880 (±120) is comparable to the ratio for 50 mmol divided by the tracer concentration of 0.003 mmol (=1667). Hence, accumulation of MSM by the tissues in directly proportion to the concentration in the bath solution. 

The total capacity of the small intestine to absorb MSM was estimated by integrating the rate absorption of MSM at 50 mmol (12.2 nmol/mg-min; average of proximal and distal tissues) with the average weight of the small intestine (1.21 ± 0.05). The resulting capacity would allow a mouse to absorb of ~0.9 mmol/h (~0.08 g/h) or ~2 g per day for a 25 g mouse, which exceeds a dose relevant for a daily supplement. 

### 3.2. Tissue Accumulation of MSM

Based on the ^35^S measured in the homogenates, all of the tissues had accumulated ^35^S after two days of dosing. The serum had higher activity than the solid tissues (Table 2). The variation among animals may have been caused by the mice eating different amounts of the vehicle, hence ^35^S labeled MSM. The amount of activity in the serum and tissue homogenates did not increase between days 2 and 8 in any of the tissues. The transiently higher activities at days 5 are attributed to the necropsies and tissue collection occurring 18–20 h after the last dosing, whereas those at days 2 and 8 were 22–24 h after the last dose. The ^35^S activity associated with the gels increased between days 2 and 8 for the serum, liver and small intestine but not for the formed elements in the blood and the skeletal muscle.

**Figure 1:**
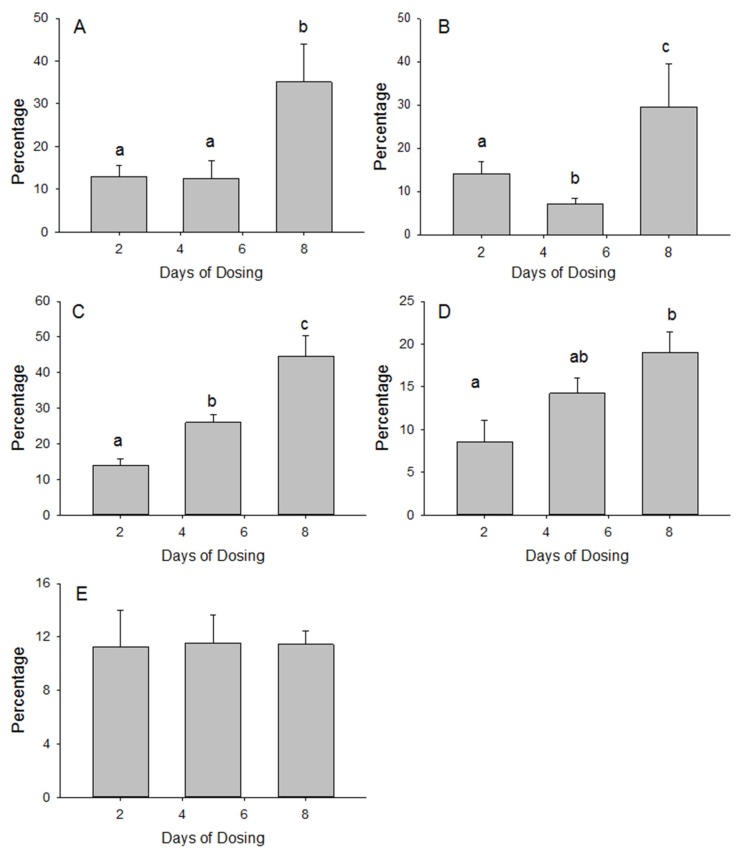
The percentages of ^35^S activity (DPM/μL) in the serum (**A**) and in homogenates of the blood cells (**B**), liver (**C**), small intestine (**D**) and skeletal muscle (**E**) of mice that were recovered in the corresponding protein gels (DPM in the gels were normalized to the volume of serum and homogenates loaded onto the gels) after supplementing the diet with ^35^S labeled MSM for 2, 5 and 8 days. Bars with different letters are significantly different (*p* < 0.05).

The percentage of ^35^S activity measured in the homogenate that was recovered in the gels was calculated to gain a better understanding of the tissue accumulation and association with the protein fraction. The percentage of liver homogenate ^35^S activity that was recovered in the gel increased more than 3-fold between days 2 and 8 of dosing (Figure 1). The serum proteins had a comparable increase in the percentage of homogenate ^35^S that was recovered in the gel. The increase in percentage recovery between days 2 and 8 was less for the blood cell fraction and small intestine but both were significant (*p* < 0.05). In contrast, the percentage of homogenate ^35^S that was recovered in the gels for the skeletal muscle remained stable at 11%.

## 4. Discussion

The present findings corroborate reports that providing a supplement of ^35^S labeled MSM results in the rapid distribution of radioactivity throughout various tissues. Studies of pharmacodynamics and tissue distribution of ^35^S reveal a rapid decline of the ^35^S after a single dose of MSM with the majority of the dose eliminated within 24 h and nearly 90% by 120 h, mainly via the urine [12,14], with more than 80% of the administered MSM having been metabolized before elimination in the urine [15]. The 8 h half-life of ingested inorganic sulfate is indicative of a similar rapid clearance [16] and is consistent with the regulation of a steady state pool of sulfate [17]. The present study provides novel findings for rapid carrier-independent, unsaturable intestinal absorption of MSM and how with chronic administration there is an increasing association of the sulfur moiety with proteins, which is consistent with how chronic feeding of MSM results in tissue retention [18].

The rapid absorption of tracer MSM by intact segments of small intestine in the presence or absence of 50 mmol unlabeled MSM indicates there is lack of competition for carriers and explains the rapid and efficient absorption in vivo [14]. The ability of MSM to passively diffuse across cell membranes is consistent with the ability to cross the blood-brain barrier [11]. In contrast, absorption of inorganic sulfate is mediated by the sodium co-transporter NaSi-1 and possibly others [17], imposing a rate-limiting step that does not apply to absorption of MSM. The capacity of the small intestine to absorb MSM greatly exceeds absorption of inorganic sulfur [19]. The resulting high MSM absorptive capacity limits availability to the bacteria in the distal small intestine and colon and contributes to the majority of ^35^S elimination via the urine, not the feces. 

The chronic provision of labeled MSM over 8 days did not result in an obvious increase in the radioactivity in the homogenates prepared from the tissues examined between days 2 and 8, implying chronic administration results in steady state levels of ^35^S in tissues. The slightly higher radioactivity associated with the tissue homogenates at day 5 was attributed to the collection of the tissues at 18 h after the previous dose rather than 24 h as at days 2 and 8. The high aqueous solubility of MSM allows for rapid, widespread and labile tissue distribution in the extracellular and cellular fluid. These same characteristics contribute to the rapid decline in the tissues after a single dose and why collection of tissues 6 h earlier would result in higher ^35^S activities. Although quantitative recovery of the ^35^S in the tissues and bedding was not done, based on previous studies, 80–90% of the administered dose would have been eliminated by 18–24 h after the last dose.

In contrast to the homogenates, for some tissues the proteins that were isolated by gel electrophoresis did exhibit increased accumulation of ^35^S between days 2 and 8 of administration. Although the specific proteins that accumulated the labeled sulfur were not identified, the increase in ^35^S provides evidence that the sulfur moiety of MSM can become associated with proteins. It is also unknown if the increasing activity in the proteins was due to incorporation of ^35^S-labeled methionine or cysteine or by posttranslational sulfation of proteins using ^35^S.

Methionine is an obligate essential amino acid and there is no known pathway of de novo synthesis for mice and other mammals. Since cysteine is derived from methionine, de novo synthesis by mice and mammals is unknown. Therefore, association of ^35^S with either methionine or cysteine would require a pathway of synthesis involving microbial metabolism. There is a complex dynamic of the co-metabolism of MSM by the microbiome and the host [20]. The bacteria in the cecum of mice and rats play an important role in nutrition. Cecal bacteria metabolize undigested feed ingredients including sulfur amino acids and host secretions, such as mucins, enzymes and sloughed cells and convert to proteins and other macro and micronutrients. Mice and rats are coprophagous and consumption of the soft stools that are produced by the cecum provide additional protein that can be digested and the constituent amino acids made available for protein synthesis [21], including synthesized sulfur amino acids [22]. This pathway is validated by the incorporation of ^35^S from MSM into the methionine isolated from plasma proteins [12]. However, because MSM is rapidly absorbed by the small intestine, the amount of MSM and labeled sulfate directly available to the bacteria is likely limited. Instead, if ^35^S from MSM is incorporated into the mucins that are secreted by the intestine [23], this would provide an alternative mechanism whereby the ^35^S would be available to the bacteria.

Posttranslational sulfation of proteins [24] provides an alternative mechanism for the gradual increase of ^35^S that was accumulated in tissue proteins. Synthesis of 3′-phosphoadenosine 5′-phosphosulfate (PAPS), the sulfonate donor for the numerous sulfotransferases involved in posttranslational sulfation and other sulfation reactions [10] requires inorganic sulfate [25]. The principal source of sulfate is considered to be cysteine, which is converted from methionine via the transulfuration pathway. Oxidation of cysteine releasing the inorganic sulfate for PAPS synthesis. However, free inorganic sulfate can also be used for sulfation of acetaminophen to reduce toxicity [26], for sulfation of mucin secreted by the intestine [27] and is incorporated into various tissues [28]. Despite a lack of designed studies, there is evidence MSM serves as a source of sulfate for synthesis of PAPS required for sulfation. Specifically, MSM contributes to the sulfation and detoxification of acetaminophen [29] and the sulfation of cartilage proteoglycans [30]. Moreover, MSM may substitute for and thereby spare cysteine and methionine as sulfate donors. Administration of MSM reduces the depletion of glutathione caused by exposure to toxins subject to sulfation for elimination [31], oxidative stress caused by acute exercise [32,33] and experimental colitis [34]. Another consideration is MSM is a safer source of sulfate when sulfur intake may be limiting compared with the sulfur amino acids, which are toxic at high levels of intake (upper safe limit of 21 mg/kg).

The different magnitudes of increase for the ^35^S isolated by gel electrophoresis of the serum and tissue homogenates are of interest but unexplained. The specific proteins that accumulated the ^35^S were not identified and it was not determined if the ^35^S was associated with incorporation of labeled methionine or cysteine or was a product of posttranslational sulfation. This will be the focus of future work.

## 5. Conclusions

Rapid intestinal absorption of MSM and penetration into tissues provides a source of sulfate for metabolism. Chronic administration of MSM does not markedly increase total tissue levels of ^35^S but some tissues exhibit a progressive increase in the amount of ^35^S that is associated with proteins; whether non-protein molecules show a similar pattern of increasing accumulation was not determined. The ^35^S associated with proteins can be explained by incorporation of bacterial synthesized methionine labeled with ^35^S or by posttranslational sulfation of proteins using ^35^S from MSM as the source of inorganic sulfate for synthesis of PAPS, which would also be involved in sulfation of other non-protein molecules. The contribution of MSM as a source of sulfate for sulfation may help explain the widespread health benefits observed with MSM supplementation. 

## Figures and Tables

**Table 1:** Rates of methylsulfonylmethane (MSM) absorption (nmol/mg-min) by the proximal and distal small intestine when measured at MSM concentrations of 50 mmol and tracer. Values are means and stnadare error of the mean.

Region	50 mmol	Tracer (0.03 mmol)
Proximal	12.7 ± 0.6	0.0063 ± 0.0004
Distal	11.6 ± 0.7	0.0068 ± 0.0005
*p* values for region comparisons	0.30	0.52

**Table 2:** Radioactivity (disintegrations per min/μg of protein) associated with serum and selected tissues of mice provided a supplement of MSM labeled with ^35^S for 2, 5 and 8 days. Values are means and SEM for total radioactivity measured in the homogenates and proteins associated with the gels after electrophoresis. Values with different letter superscripts in the same column for the homogenates and gels are significantly different (*p* < 0.05).

Days	Serum	Blood Cells	Liver	Small Intestine	Skeletal Muscle
	Homogenate radioactivity				
2	140.1 ± 17.7 ^a^	0.98 ± 0.42 ^ab^	2.70 ± 1.03	4.31 ± 1.65 ^ab^	3.38 ± 1.13
5	132.2 ± 11.4 ^a^	1.50 ± 0.29 ^b^	3.22 ± 0.47	5.77 ± 0.42 ^a^	4.65 ± 0.73
8	70.9 ± 12.8 ^b^	0.78 ± 0.12 ^a^	2.78 ± 0.24	3.71 ± 0.39 ^b^	3.47 ± 0.47
	Radioactivity in the gel				
2	8.55 ± 1.20 ^a^	0.14 ± 0.06	0.14 ± 0.07 ^a^	0.27 ± 0.05 ^a^	0.31 ± 0.07 ^a^
5	7.99 ± 2.11 ^a^	0.10 ± 0.02	0.55 ± 0.07 ^b^	0.79 ± 0.08 ^b^	0.48 ± 0.02 ^b^
8	11.02 ± 2.12 ^b^	0.24 ± 0.11	0.81 ± 0.07 ^c^	0.71 ± 0.12 ^b^	0.39 ± 0.04 ^ab^
